# Defective apoptotic cell clearance activates innate immune response to protect *Caenorhabditis elegans* against pathogenic bacteria

**DOI:** 10.1080/21505594.2020.1857982

**Published:** 2020-12-29

**Authors:** Jinlong Wan, Lei Yuan, Huiru Jing, Qian Zheng, Hui Xiao

**Affiliations:** Key Laboratory of the Ministry of Education for Medicinal Plant Resources and Natural Pharmaceutical Chemistry, National Engineering Laboratory for Resource Development of Endangered Crude Drugs in the Northwest of China, College of Life Sciences, Shaanxi Normal University, Xi’an, China

**Keywords:** *C. elegans*, apoptosis, apoptotic cell clearance, innate immune, lysosomes

## Abstract

Appropriate clearance of dead cells generated by apoptosis is critical to the development of multicellular organisms and tissue homeostasis. In mammals, the removal of apoptotic cell is mediated by polarized monocyte/macrophage populations of the innate immune system. The innate immune system is essential for anti-viral and anti-microbial defense. However, our current understanding of the relationship between apoptotic cell clearance and the innate immune response has remained rather limited. Here, we study how apoptotic cell clearance programs contribute to the innate immune response in *C. elegans*. We find apoptotic cell clearance mutant worms are more resistant to pathogenic bacteria of *Pseudomonas aeruginosa* PA14 and *Salmonella typhimurium* SL1344 due to significant upregulation of innate immune-dependent pathogen response genes. In addition, genetic epistasis analysis indicates that defects in apoptotic cell clearance can activate the innate immune response through PMK-1 p38 MAPK and MPK-1/ERK MAPK pathways in *C. elegans*. Taken together, our results provide evidence that insufficient clearance of apoptotic cell can protect *Caenorhabditis elegans* from bacterial infection through innate immune response activation.

## Introduction

Apoptosis is the major process of programmed cell death and is evolutionarily conservative, which eliminates abnormal or damaged cells during development [1,2]. The appropriate clearance of apoptotic cell is indispensable for apoptosis and is crucial for the development of multicellular organisms and homeostatic [3]. If apoptotic cell cannot be removed in time and effectively, the harmful contents released by the apoptotic cell will lead to inflammation or autoimmune diseases, such as systemic lupus erythematosus and rheumatoid arthritis [4].

*Caenorhabditis elegans* has been used as an excellent model organism for studying the mechanism of apoptotic cell (corpse) clearance. *C. elegans* lacks any “professional” phagocytes, corpses are quickly engulfed and degraded by neighboring cells [5]. When cells undergo apoptosis, Caspase-mediated activation of CED-8 promotes phosphatidylserine (PS) externalization in apoptotic cell, which is an “eat-me” signal exposed on the surface of apoptotic cell and recognized by phagocytes [6]. The engulfment of apoptotic cell is mainly accomplished by two parallel redundant genetic pathways that are highly evolutionarily conserved [4,7]. In the *ced-1/6/7* pathway, the phagocytic receptor CED-1 recognizes PS [8]. Then CED-6 interacts with CED-1 to transduce engulfment signals to downstream effectors. CED-7, an ABC transporter homolog, can expose PS to the surface of apoptotic cell [5,9]. In the *ced-2/5/12* pathway, CED-2, an adaptor protein containing SH2 and SH3 domains, activates the CED-5/CED-12 complex [5,9]. The CED-5/CED-12 complex acts as a bipartite nucleotide exchange factor to activate Rac GTPase CED-10, which in turn causes rearrangement of the actin cytoskeleton for cell corpse engulfment [5,9]. The *ced-1/6/7* and *ced-2/5/12* pathways lead to the internalization of apoptotic cell and the formation of phagosomes [7]. Then, the phagosomes are fused with lysosomes to form phagolysosomes. In this process, VPS-18 is necessary for the endosome and lysosome biogenesis and fusion of phagosome and lysosome [10]. Finally, the corpses are digested by lysosomal acid hydrolases in the phagolysosome, such as NUC-1, a *C. elegans* DNase II homolog [11].

Besides apoptotic cell clearance, studies have demonstrated a certain evolutionary conservation between the mammalian innate immune and that of invertebrates [12,13]. The invertebrate *C. elegans* relies entirely on its innate immune to defend against pathogens, has been widely used to study the interaction between host and pathogen. For example, the human opportunistic pathogens can establish persistent infection in the intestine of *C. elegans* [14–16]. Researches on nematodes infected with various pathogens help people understand the mechanism of innate immunity [16]. The innate immunity in *C. elegans* is regulated by several major pathways to defense against pathogens including PMK-1 p38 MAPK pathway and MPK-1/ERK MAPK pathway [16,17]. In the PMK-1 pathway, NSY-1 phosphorylates SEK-1, SEK-1 phosphorylates PMK-1, and finally activates PMK-1. In the MPK-1 pathway, LIN-45 phosphorylates MEK-2, MEK-2 phosphorylates MPK-1, and finally activates MPK-1. Studies have shown that activation of the MPK-1/ERK MAPK pathway in *C. elegans* can facilitate the germ cells apoptosis, depending at least in part on the phagocytic mechanism of apoptotic cell [17]. However, little is known about how apoptotic cell clearance affects innate immunity in *C. elegans.*

In mammals, apoptotic cell clearance is mainly mediated by professional phagocytes of innate immune system [18]. It has been demonstrated that apoptotic cell can be engulfed by macrophages in DNase II-knockout mice, but the DNA of the dead cells is not properly degraded in the lysosomes, which activate the innate immune response leading to severe anemia and chronic arthritis [19]. Similarly, antibacterial peptide genes are expressed when fragmented DNA is not cleared from apoptotic germ cells by loss of DNase II in Drosophila and *C. elegans* [20,21]. However, evidence from other studies suggests that DNase II deficiency impairs innate immune function in Drosophila [22]. Studies have also shown that regulation of unfolded protein response by CED-1 is essential for innate immunity in *C. elegans* [23]. Thus, there are still a lot of work required to determine the relationship between apoptotic cell clearance and innate immunity.

In the present study, we systematically investigate the relationship between apoptotic cell clearance and innate immunity in *C. elegans*. We found that defective apoptotic cell clearance can trigger the innate immune response of *C. elegans* through PMK-1 p38 MAPK and MPK-1/ERK MAPK pathways, which protect worms against pathogenic bacteria infection. Our results provide an important basis for further elucidating the underlying mechanism of how clearance of apoptotic cell regulates innate immune responses.

## Materials and methods

### C. elegans *and bacterial strains*

*C. elegans* maintenance was performed using standard protocols [24]. The strains used in this study were mostly obtained from the Caenorhabditis Genetics Center, which is funded by the NIH Office of Research Infrastructure Programs (P40 OD010440).

### RNA interference (RNAi)

RNA interference was performed by feeding *C. elegans* with RNAi bacteria that express double-stranded RNA (dsRNA) targeting the gene of interest. 129.36 RNAi, *ced-1* RNAi, *ced-2* RNAi, *nsy-1* RNAi, *pmk-1* RNAi, *mpk-1* RNAi were obtained from the RNAi library. The *tir-1* RNAi and *sek-1* RNAi were provided by our laboratory.

### Lifespan assays

Lifespan assays by *P. aeruginosa* PA14 infection were conducted at 25°C [25]. Lifespan assays by *S. typhimurium* SL1344 infection were conducted at 20°C [15]. A total of 100 worms were quantified in each lifespan assay, and all experiments were repeated at least three times. Detailed procedures are provided in Supplementary Materials and methods.

### Real-time quantitative RT-PCR (qRT-PCR) assays

Total RNA was extracted using the Trizol method. cDNA was synthesized using Transcriptor First Strand cDNA Synthesis Kit (Roche). Quantitative real-time PCR was performed using a StepOne Plus Real-Time PCR system and SYBR qPCR Master Mix (Vazyme). RNA fold change was calculated by comparing mRNA levels of the gene of interest with mRNA levels of the reference gene *tbg-1*. The primers used in this study are listed in Supplementary Table S6.

### Fluorescence microscopy

For imaging fluorescence, worms were mounted onto 2% agar pads, paralyzed with levamisole. The slides were viewed using fluorescence microscope (Zeiss M2) and processed with ImageJ (http://rsb.info.nih.gov/ij/).

### Immunoblot analyses

Protein quality was determined by Immunoblot analysis. Detailed procedures are provided in Supplementary Materials and methods.

### Statistical analysis

Statistical analysis was performed with the two-tailed student’s t-test with GraphPad Prism 8 software. Values of *P* < 0.05 were considered statistically significant.

## Results

### *Defective clearance of apoptotic cell in* C. elegans *enhanced resistance to infection by* P. aeruginosa *PA14 and* S. typhimurium *SL1344*

**Figure 1.**
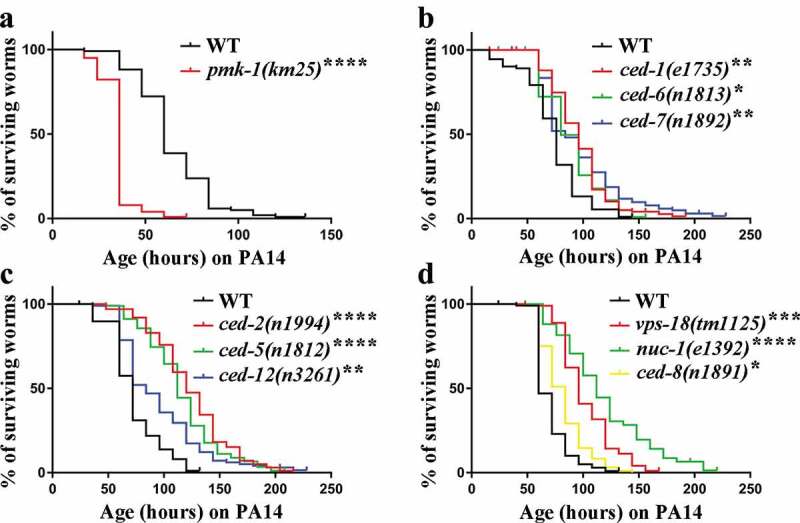
Defective clearance of apoptotic cell extends *C. elegans* lifespan on *P. aeruginosa* PA14. (a-d) Lifespan analyses in the indicated strains. Two-tailed student’s t-test method was performed to compare all the other datasets with wild type (WT). See Supplementary Table S1 for detailed statistical analysis of lifespan data. *, *P* < 0.05; **, *P* < 0.01; ***, *P* < 0.001; ****, *P* < 0.0001. Error bars represent SEM

To examine the interaction between apoptotic cell clearance and innate immune response in *C. elegans*, we used apoptotic cell clearance defective mutant nematodes and analyzed their resistance to pathogenic bacteria. As expected, when infected by *P. aeruginosa* PA14 and *S. typhimurium* SL1344, the lifespans of *pmk-1(km25)* mutants were significantly reduced compared to the wild-type N2 (Figure 1(a) and S1A; Table S1 and S2) [26,27]. Next, we conducted lifespan analysis on strong loss-of-function mutants of *ced-1/6/7* and *ced-2/5/12* pathway components and found that lifespans of these engulfment mutants were significantly extended than N2 after *P. aeruginosa* PA14 and *S. typhimurium* SL1344 infection (Figure 1(b and c); Figure S1B and S1C; Table S1 and S2). To test whether resistance to pathogenic bacteria infections was limited to engulfment mutants, we performed lifespan analysis on *ced-8(n1891), vps-18(tm1125)*, and *nuc-1(e1994*), and observed that these mutants lifespans were also significantly extended (Figure 1(d) and S1D; Table S1 and S2). Moreover, the lifespan of the *ced-1(e1735); ced-2(n1994)* double mutant feeding on SL1344 but not on PA14 was significantly extended when compared to the single mutant (Figure S2B). Furthermore, using a GFP expressing PA14(PA14-GFP), we examined bacterial accumulation in the intestine of worms, and found no significant difference between *ced-1(e1735), ced-2(n1994), ced-8(n1891),* and wild-type N2 (Figure S3A-C). To corroborated the fluorescence data, we performed colony-forming units (CFUs) experiment and found there was more bacterial accumulation in the intestine of *ced-2(n1994)* and *ced-8(n1891)* but not in *ced-1(e1735*), which indicate apoptotic cell clearance does not block the accumulation of *P. aeruginosa* PA14 in the intestine. These results demonstrate that apoptotic cell clearance defective mutants are resistant to infection by pathogenic bacteria.

### *Defects in apoptotic cell clearance upregulate defense gene expression in* C. elegans

**Figure 2.**
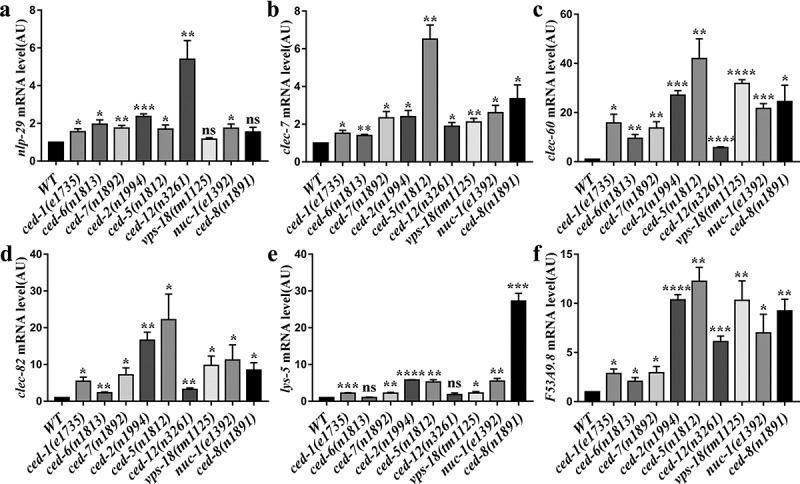
Increased expression of pathogen response genes in apoptotic cell clearance mutants without bacteria pathogens infection. (a-f) Bars represent mRNA levels for *nlp-29* (a), *clec-7* (b), *clec-60* (c), *clec-82* (d), *lys-5* (e) and *F53A9.8* (f) pathogen response genes determined by qRT-PCR in WT and apoptotic cell clearance defective mutant cultured on live OP50. Values in arbitrary units (AU) are the average of at least 3 biological replicates. *, P < 0.05; **, P < 0.01; ***, P < 0.001; ****, P < 0.0001; not significant (ns), *P > 0.05*. Two-tailed Student’s t-test. Error bars represent SEM

*C. elegans* does have an innate immune system and responds to pathogenic bacterial by expression of defense genes [28]. To test whether defects in apoptotic cell clearance activate the innate immune response in *C. elegans*, we analyzed the mRNA level of pathogen response genes by Real-time quantitative RT-PCR (qRT-PCR). In *C. elegans, clec-7, clec-60*, and *clec-82* encode C-type lectin proteins, *lys-5* encodes lysozyme, *nlp-29* and *F53A9.8* encode antimicrobial peptides [29,30]. Intriguingly, we observed that not all but most of the pathogen response genes listed above were upregulated in apoptotic cell clearance mutants without pathogenic bacterial infections (Figure 2). The expression of NLP-29 is regulated by a conserved innate immune signaling cascade specifically in the epidermis during infection [30]. To confirm the above findings, we knocked down *ced-1* or *ced-2* by RNAi in transgenic worms carrying a *pnlp-29::gfp* and a *pcol-12::dsRed* transcriptional reporters constructs, and investigated the expression level of *nlp-29*. Transgenic worms appear predominantly red in the absence of infection due to the constitutively expressed P*_col-12_*::dsRED under the control of hypodermis-specific promoter [31]. We observed the reporter green fluorescent protein (GFP) fluorescence in transgenic worms was notably increased by RNAi treatment of *ced-1* or *ced-2* without bacterial pathogens infections, which colocalized with P*_col-12_*::dsRED (Figure S4 A and B). The increased level of P*_nlp-29_*::GFP in transgenic worms was confirmed by Western blotting (Figure S4 C and D). Taken together, these results show that the defective clearance of apoptotic cell results in upregulation of pathogen defense genes in the absence of pathogenic bacterial infections in *C. elegans*.

### *Apoptotic cell clearance deficiency activates PMK-1 p38 MAPK and MPK-1/ERK MAPK pathways in* C. elegans

**Figure 3.**
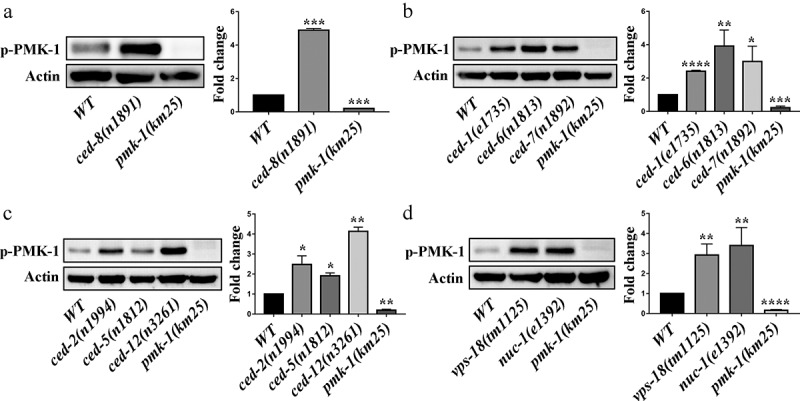
Defective clearance of apoptotic cell increases the level of activated PMK-1 in *C. elegans*. (a-e) Immunoblot analysis of lysates from worms using antibodies that recognize PMK-1 (p-PMK-1) and Actin (loading control). Worms at the L4 stage were cultured on plates containing live OP50 for approximately 48 hours and lysates were prepared. The blot is typical of three independent experiments. *, P < 0.05; **, P < 0.01; ***, P < 0.001; ****, P < 0.0001. Two-tailed Student’s t-test. Error bars represent SEM

Our results in line with previous studies that loss- and reduction-of-function mutations of p38 MAPK PMK-1 pathway components lead to a reduction in the levels of activated PMK-1 protein (phosphorylated PMK-1) [26,32] (Figure 3). To explore whether apoptotic cell clearance participates in the innate immune response through the PMK-1 p38 MAPK and MPK-1/ERK MAPK pathways, we measured the expression level of PMK-1 and MPK-1 in apoptotic cell clearance mutant worms. We found there is no significant difference between PMK-1 and MPK-1 in these mutants by using actin as loading control (Figure S5). We then measured activated PMK-1 and MPK-1 levels by immunoblotting in *C. elegans*. Our results showed that both activated PMK-1 and activated MPK-1 in apoptotic cell clearance defective mutants were significantly increased compared to wild-type controls (Figure 3 and Figure S6). To further confirm PMK-1 and MPK-1 pathways were activated in apoptotic cell clearance mutants, we detected the expression level of *irg-5* and *sysm-1*, which are used as canonical PMK-1 effector reporters [33,34], and detected the expression level of *mpk-1* dependent genes *lin-39* and *egl-5* [35,36]. We found *irg-5, sysm-1, lin-39*, and *egl-5* were upregulated in most of apoptotic cell clearance mutant worms (Figure S7). To test whether generic stress is involved in apoptotic cell clearance mutants against pathogens, we detected the expression levels of *prdx-3* and *lgg-1*, the endogenous targets for DAF-16 in *ced-1* and *ced-2* mutants [37], and found no significant difference when compared to wild type (Figure S8). These data prove that defects in apoptotic cell clearance can activate PMK-1 p38 MAPK and MPK-1/ERK MAPK pathways in *C. elegans*, suggesting that defective apoptotic cell clearance may be involved in the regulation of innate immune response through the PMK-1 p38 MAPK and MPK-1/ERK MAPK pathways.

### PMK-1 p38 MAPK and MPK-1/ERK MAPK pathways are required for the resistance of apoptotic cell clearance mutants against pathogens

**Figure 4.**
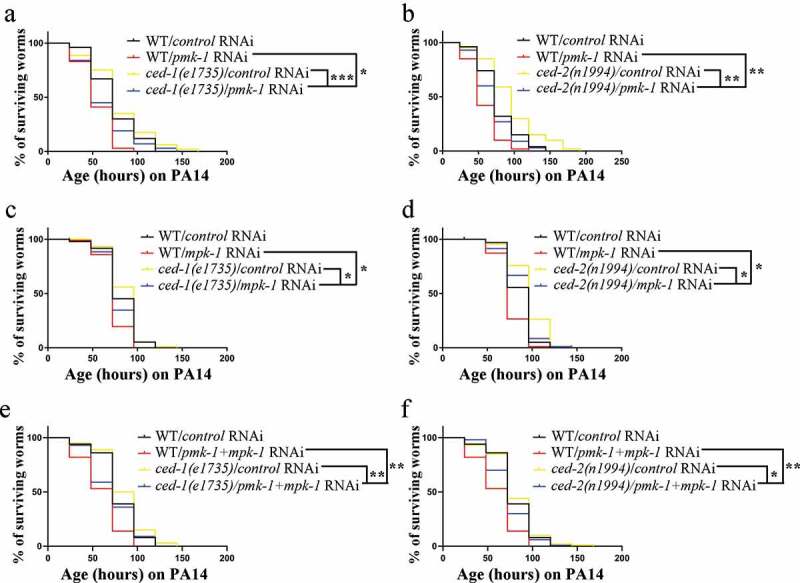
*Pmk-1* and *mpk-1* are required for lifespan extension of *ced-1(e1735)* and *ced-2(n1994)* mutants after exposure to *P.aeruginosa* PA14. (a-f) Lifespan analyses in the indicated strains. *ced-1(e1735)* (a), (c), (e)and *ced-2(n1994)* (b), (d), (f) were fed the indicated RNAi bacteria and transferred at the L4 stage to plates containing *P. aeruginosa* PA14 bacteria. Two-tailed student’s t-test method was performed to compare all the other datasets with wild type N2 (WT). All lifespan assays were carried out at 25°C and were repeated at least three times. *, P < 0.05; **, P < 0.01; ns, P > 0.05.

To better define how apoptotic cell clearance participates in the innate immune response through the MPK-1/ERK MAPK pathway, we measured the lifespan of *ced-1(e1735)* and *ced-2(n1994)* mutants infected by *P. aeruginosa* PA14. The *ced-1 (e1735)* and *ced-2(n1994)* mutants showed the significantly reduced lifespan by *pmk-1* RNAi and *mpk-1* RNAi treatment, respectively (Figure 4; Table S3 and Table S4). These imply that *pmk-1* and *mpk-1* are required for *ced-1 (e1735)* and *ced-2(n1994)* mutants lifespan extension after *P. aeruginosa* PA14 infection. Moreover, we found *pmk-1* RNAi or *mpk-1* RNAi treatment partially suppressed the extended lifespan phenotype of *ced-1(e1735)* mutants as well as *ced-2(n1994)* mutants when compared to wild-type control (Figure 4; Table S3 and Table S4), which indicate there is functional redundancy among p38 MAPK and MPK-1 MAPK for apoptotic cell clearance mutants against pathogens. To test whether there is functional redundancy among p38 MAPK and MPK-1 MAPK for apoptotic cell clearance mutants against pathogens, we performed the *mpk-1(RNAi);pmk-1(RNAi)* double knockdown in *ced-1(e1735)* and *ced-2(n1994)* backgrounds. We found *mpk-1;pmk-1* double RNAi treatment still partially suppressed the extended lifespan phenotype of *ced-1(e1735)* mutants as well as *ced-2(n1994)* mutants when compared to wild-type control (Figure 4; Table S5), which indicates other innate immune pathways are involved in protecting apoptotic cells clearance mutants against pathogen. However, we can’t exclude that the redundancy between these two MAPK pathways, as double feeding is less reliable than single feeding. In some cases, only one gene may be significantly inhibited, or both genes may be only slightly knocked down. While our genetic epistasis analysis suggested that *ced-1* and *ced-8* act upstream or parallel to *nsy-1*, while *ced-2* acts upstream or parallel to *pmk-1* to regulate the PMK-1 p38 MAPK pathway (Figure S9). Therefore, we propose that apoptotic cell clearance defects actively regulate the innate immune response through the PMK-1 p38 MAPK and MPK-1/ERK MAPK pathways in *C. elegans*.

## Discussion

Apoptotic cell clearance in *C. elegans* can be divided into three main steps: recognition of corpse, corpse internalization/engulfment, and corpse degradation [5]. Here, we show that defects in these three key steps of apoptotic cell clearance can protect worms against infection by pathogenic bacterial *P. aeruginosa* PA14 and *S. typhimurium* SL1344. We observed an accumulation of GFP-expressing PA14 in the intestine of *ced-1(e1735)* and *ced-2(n1994)* mutants. These suggest the protective effect of engulfment genes loss of function is not caused by inadequate phagocytosis of bacterial pathogens. In addition, we identify that defective clearance of apoptotic cell activates the innate immune response through conserved PMK-1 p38 MAPK and MPK-1/ERK MAPK pathways in *C. elegans*.

We found defects in apoptotic cell clearance upregulated innate immune response gene expression in the absence of bacterial pathogens in *C. elegans*. Despite lacking both Toll and Imd pathways as well as the immunological memory of vertebrate adaptive immunity, *C. elegans* is considered to have an innate immune defense system and respond to pathogen infection by expression of antimicrobial peptides [31]. In mammals, the swift and efficient clearance of apoptotic cell by professional phagocytes is crucial to avoid the loss of plasma membrane integrity and release of cellular contents to prevent unwanted immune responses to self-antigens that are derived from these dying cells [18]. On the other hand, macrophages that ingested apoptotic cell secrete anti-inﬂammatory cytokines and actively suppress the secretion of pro-inflammatory cytokines [38]. Apoptotic cell digestion in lysosomes has long been the least considered stage, possibly because it was believed to function as garbage disposal. However, recent studies have shown that macrophages lacking lysosome DNase II activated innate immunity via a STING-dependent pathway leading to severe anemia and chronic polyarthritis [39]. Innate immunity activation has also been detected in DNase II-deficient flies and worms which constitutively expressed antibacterial peptides genes [20,21]. Our results are consistent with the above-described finding that pathogen response genes were upregulated in *nuc-1(n1994)* mutants without pathogenic bacterial infections. NUC-1 is implicated in removing apoptotic DNA and in digesting ingested bacterial DNA in the intestine in *C. elegans* [11]. Upregulation of pathogen response in *nuc-1(n1994)* mutants was likely not due to failure to digest ingested bacterial DNA, First, similar results have been found in engulfment mutant, apoptotic cell PS externalization mutant, and apoptotic cell degradation mutant, indicating the general effects in upregulating innate immune response gene expression by defects in apoptotic cell clearance in *C. elegans*. Second, study has shown that loss of DNase II function in other tissues such as the gonad is associated with increased expression of antimicrobial genes [20]. In *C. elegans*, cell corpses are rapidly removed by neighboring cells such as hypodermal cells, muscle cells, intestinal cells, and sheath cells [7]. It will be necessary to study when and which cells express the antibacterial peptide genes in apoptotic cell clearance-deficient worms.

We found defective clearance of apoptotic cell activated PMK-1 p38 MAPK and MPK-1/ERK MAPK pathways but not the DAF-16 pathway in *C. elegans*. Furthermore, we demonstrated that PMK-1 p38 MAPK and MPK-1/ERK MAPK pathways were required for apoptotic cell clearance mutants against pathogens. In *C. elegans*, the expression level of *nlp-29* is regulated in the epidermis by the innate immune signaling cascade, consisting of TIR-1/SARM, NSY-1/MAP3K, SEK-1/MAP2K, and PMK-1/p38 MAPK [31]. We observed the upregulation of *nlp-29* expression in apoptotic cell clearance mutant worms. In line with these results, our immunoblotting experiments showed that activated PMK-1 and MPK-1 in apoptotic cell clearance-deficient nematodes were significantly increased compared to wild-type controls. Meanwhile, our lifespan analysis showed that *ced-1(e1735)* and *ced-2(n1994)* mutants exhibited enhanced susceptibility to *P. aeruginosa* PA14 infection after knockdown of *pmk-1* or *mpk-1* when compared to control RNAi. These indicate *pmk-1* and *mpk-1* are essential for apoptotic cell clearance mutants against pathogens. Further studies will be needed to clarify whether other innate immune pathways are involved in protecting apoptotic cell clearance mutants against pathogens. Moreover, by genetic epistasis analysis, we found that *ced-1* and *ced-8* act upstream or parallel to *nsy-1*, while *ced-2* acts upstream or parallel to *pmk-1* to regulate the p38 MAPK pathway. As the *ced-1(e1735);ced-2(n1994)* double mutants were more resistant to bacterial pathogens SL1344 but not PA14 compared to the single mutants. We proposed that *ced-1* and *ced-2* may function redundantly in the regulation of p38 MAPK pathway against SL1344. Further research should be undertaken to investigate how apoptotic cell clearance regulates innate immune responses in *C. elegans*. However, the findings of the current study are different from the previous research that unfolded protein response genes regulated by CED-1 and loss of function of *ced-1* lead to compromised innate immune response in *C. elegans* and are rapidly killed by live bacteria [23,40]. There are possible explanations for this might be that they performed *C. elegans* killing assay by *S. typhimurium* at 25°C [23] instead of at 20°C and use different *S. typhimurium* strain [40]. At a higher temperature, *S. typhimurium* may grow faster to colonize and proliferate in the worm intestine, which likely masks the protective effect of *ced-1* loss of function. Another possible explanation for this is that high temperatures are known to induce unfolded protein response. Unfolded protein response may become more important for innate immune response in *C. elegans* at a higher temperature. During apoptotic cell clearance, CED-1 is internalized from and recycled back to the cell membrane. Previous study demonstrated that loss of retromer function results in lysosomal accumulation of CED-1 [41]. It is also interesting to test whether retromer would affect innate immunity in *C. elegans*. Future studies on determining the mechanisms of current differences are therefore recommended.

In summary, our results provide evidence that defective apoptotic cell clearance can actively regulate the innate immune response through PMK-1 p38 MAP\K and MPK-1/ERK MAPK pathways in *C. elegans*, establish the link between the apoptotic cell clearance and the innate immune response. Inefficient clearance of dead cells activates the innate immune system that has been documented in mammalian [19]. Thus, our research provides important clues for further dissecting the underlying mechanism of how apoptotic cell clearance regulates innate immune responses.

## Supplementary Material

Supplemental Material
