# Supplementary material for: Defective apoptotic cell clearance activates innate immune response to protect Caenorhabditis elegans against pathogenic bacteria
Source: Virulence. 2020 Dec 29;12(1):75–83. doi: 10.1080/21505594.2020.1857982 (PMC7781629; doi:10.1080/21505594.2020.1857982)
Supplement: Supplemental Material [file KVIR_A_1857982_SM0554.docx]

**Supplemental Information：**

**Defective apoptotic cell clearance active innate immune response to protect *Caenorhabditis elegans* against pathogenic bacteria**

Jinlong Wan, Lei Yuan, Huiru Jing, Qian Zheng, Hui Xiao


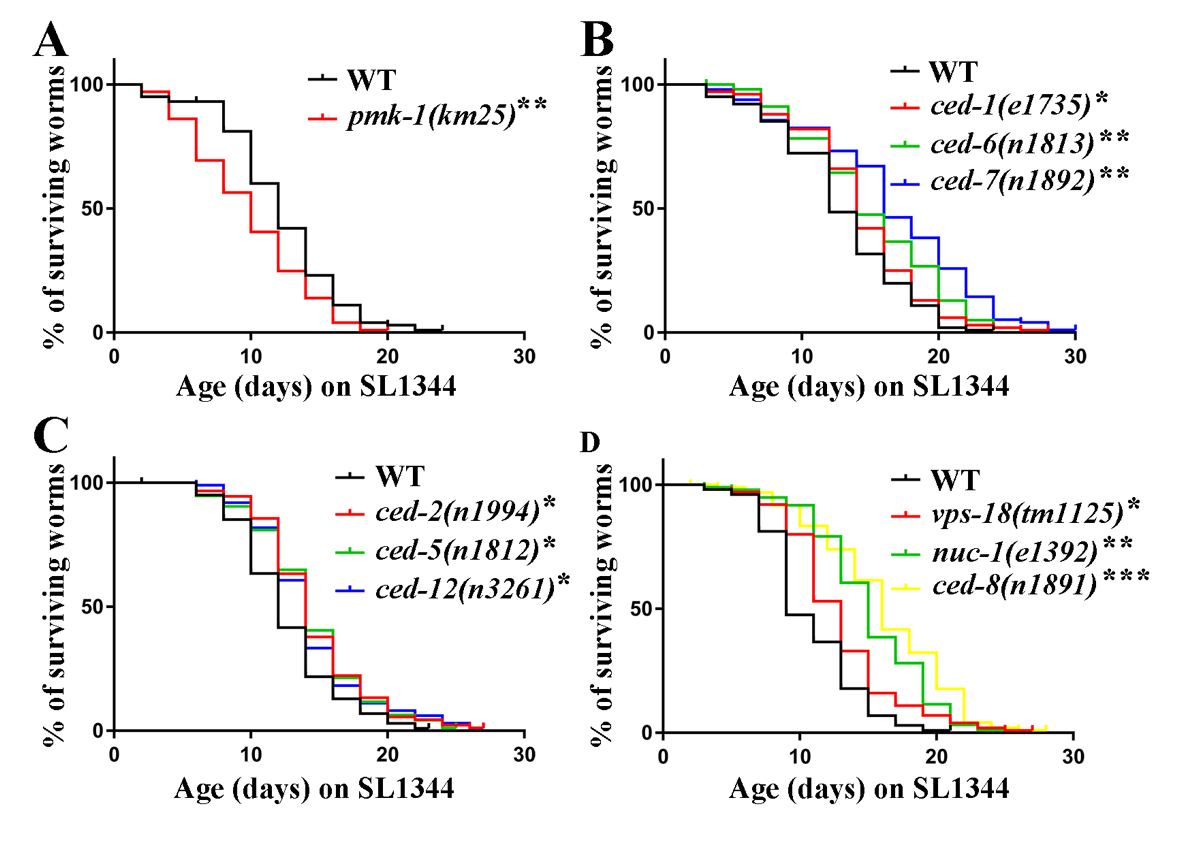


Figure S1 Defective clearance of apoptotic cell extends *C. elegans* lifespan on *S. typhimurium* SL1344.Related to Figure1

(A-D) Lifespan analyses in the indicated strains. A total of 100 worms were quantified in each strain. Two-tailed student's t-test method was performed to compare all the other datasets with wild type (WT). All lifespan assays were carried out at 20 °C and were repeated at least three times. Please see Supplementary Table S2 for detailed statistical analysis of lifespan data. *, *P* < 0.05; **, *P* < 0.01; ***, *P* < 0.001. Error bars represent SEM.


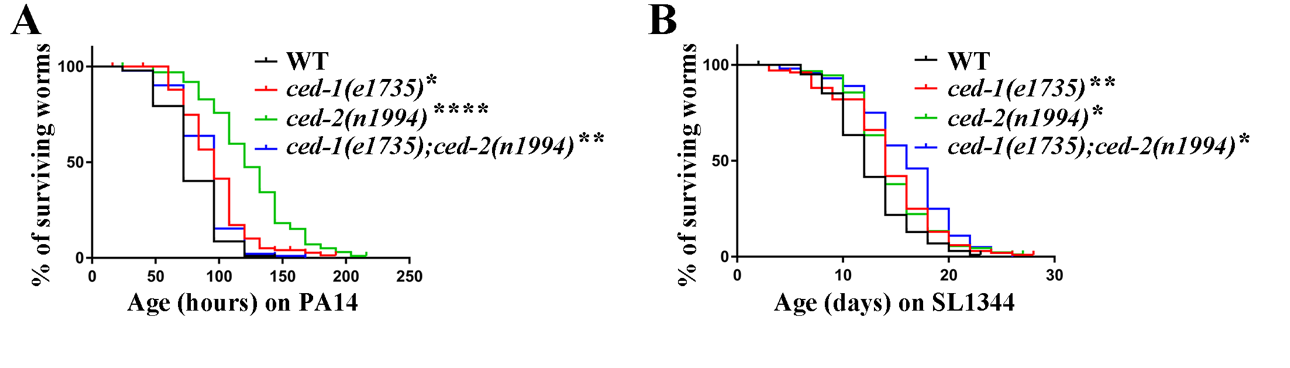


Figure S2 *ced-1* and *ced-2* double loss-of-function mutants are more resistant to infection by SL1344. Related to Figure1

(A) Lifespan analysis of the indicated *P. aeruginosa* PA14 strains. The lifespan assays were carried out at 25 °C. (B) Lifespan analysis of the indicated *S. typhimurium* SL1344 strains. The lifespan assays were carried out at 20 °C. A total of 100 worms were quantified in each strain. Two-tailed student's t-test method was performed to compare all the other datasets with wild type (WT). All lifespan assays were repeated at least three times. *, *P* < 0.05. Error bars represent SEM.


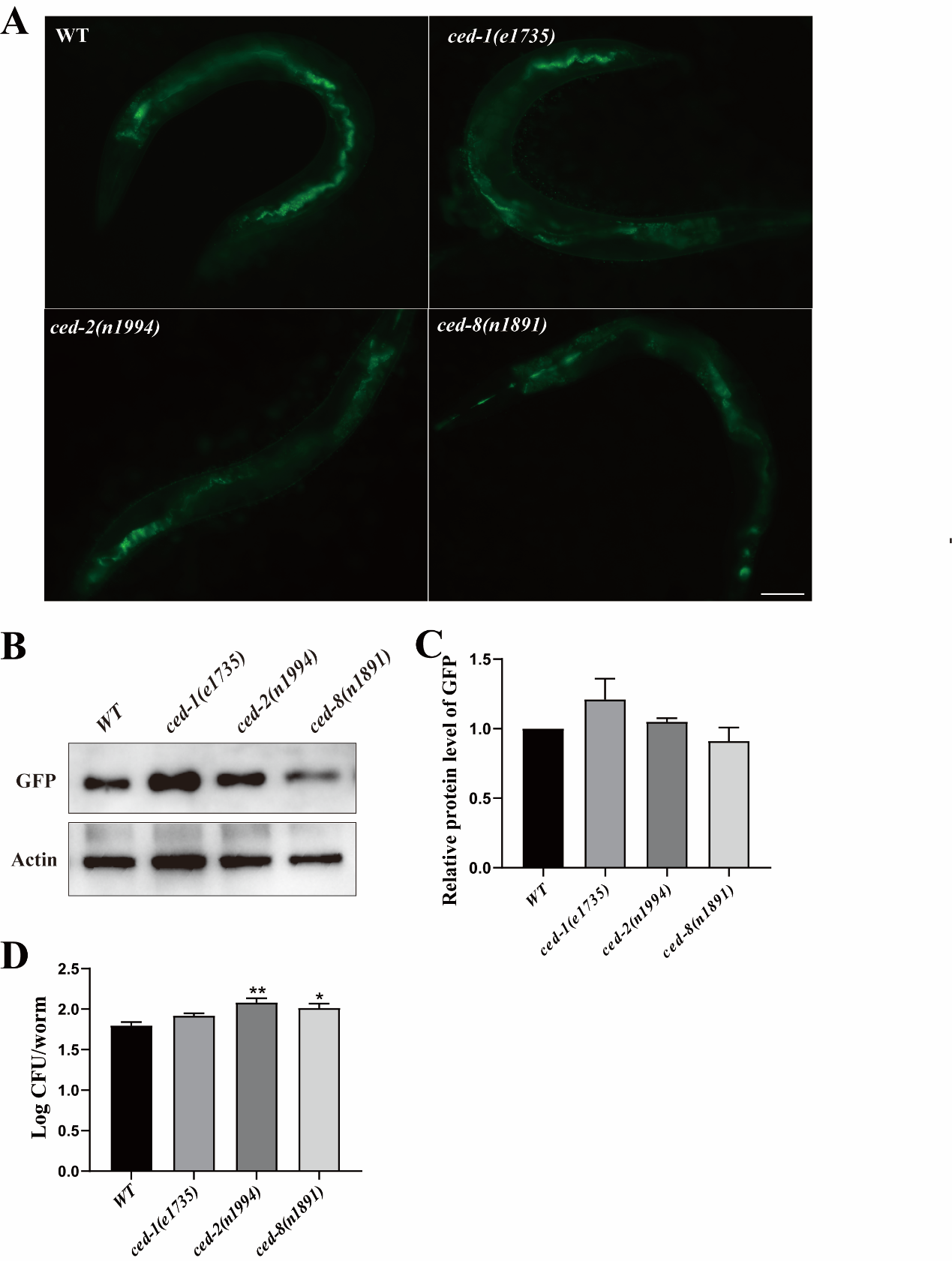


Figure S3 Apoptotic cell clearance does not block the accumulation of *P. aeruginosa* PA14 in the intestine. Related to Figure1

(A)The GFP fluorescence image of the entire lumen in wild type and corpse clearance mutants fed on *P. aeruginosa* PA14-GFP for 48 h. Bars, 50 µm. (B) Immunoblot test of lysates from worms of the indicated genotype using antibodies that recognize GFP (anti-GFP) and Actin (loading control). (C) Quantitative analysis of GFP expression levels. Two-tailed Student’s t-test. (D) Colony forming unit of wild type and corpse clearance mutants exposed to *P. aeruginosa* PA14 at 48h. Data are presented as mean±SEM of four independent biological replicates


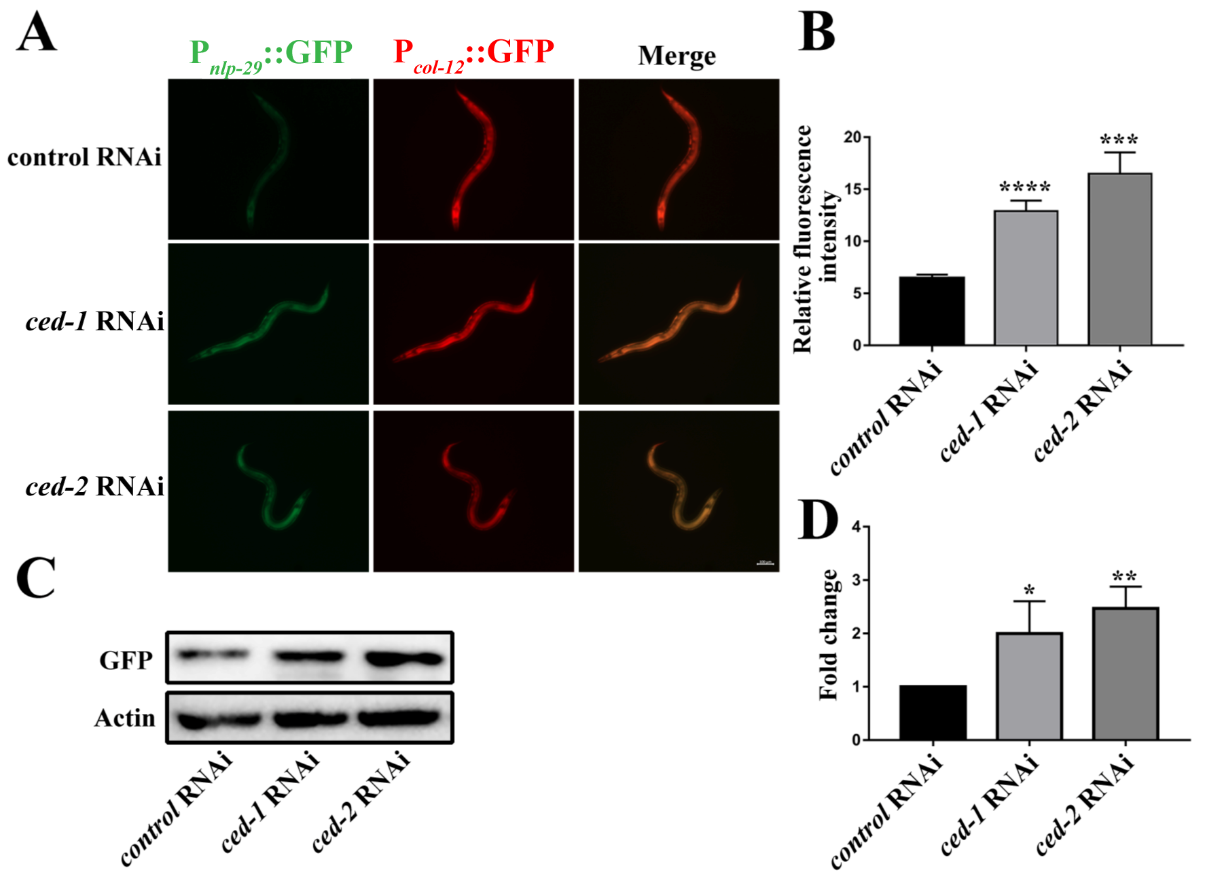


Figure S4 RNAi knock-down of *ced-1* and *ced-2* increase the expression level of *nlp-29.* Related to Figure 2

(A) Fluorescent images of the hypodermis in the transgenic worm HMZ009 expressing P*_nlp-29_*::GFP. HMZ009 were fed the indicated RNAi bacteria and transferred at the L4 stage to plates containing RNAi bacteria for approximately 48 h. Green fluorescence is *pnlp-29::gfp* induction and red fluorescence is *pcol-12::dsRed* induction. At least 10 worms were scored in each strain. Bars, 100 µm. (B) Quantitative analysis of GFP expression levels. Two-tailed Student’s t-test. (C) Immunoblot test of lysates from worms of the indicated genotype using antibodies that recognize GFP (anti-GFP) and Actin (loading control). (D) Quantitative analysis of immunoblot test results. ***, *P* < 0.001; ****, *P* < 0.0001. Error bars represent SEM.


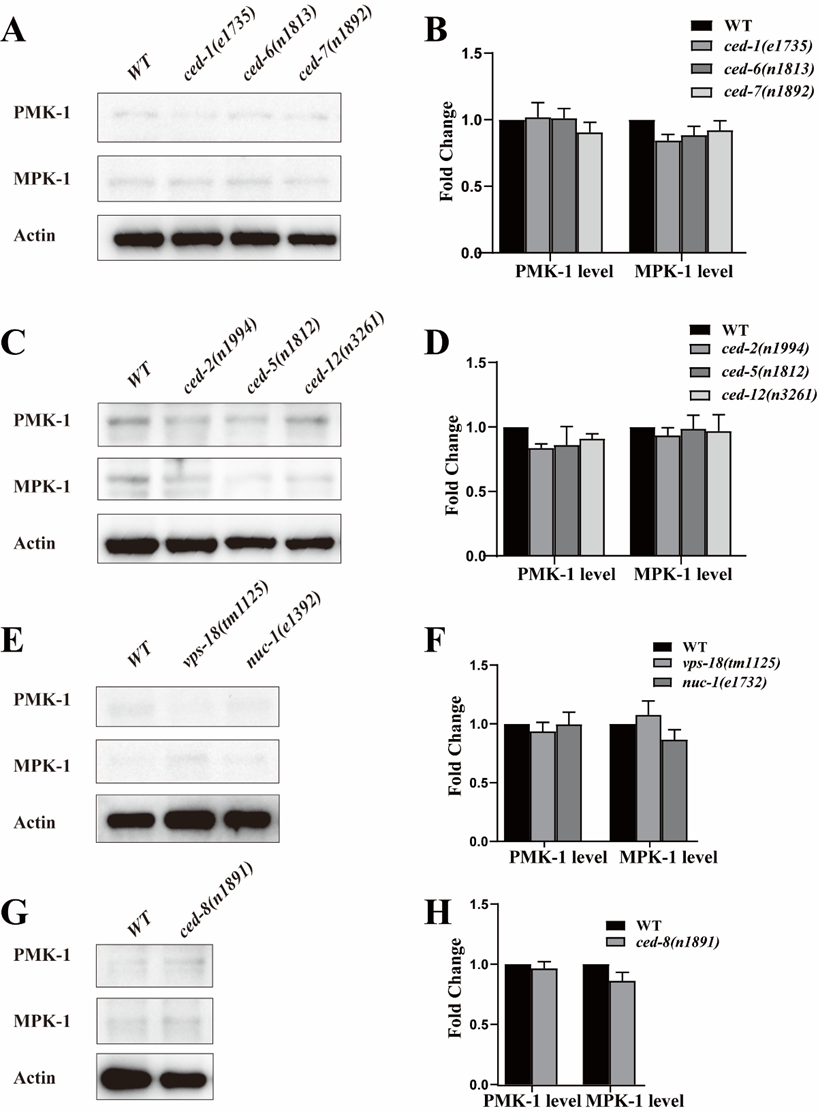


Figure S5 Defective clearance of apoptotic cell have no effect on the level of PMK-1 and MPK-1 in *C. elegans.* Related to Figure3

(A-H) Immunoblot analysis of lysates from worms of apoptotic cell clearance defective mutants and wild-type N2 (WT) using antibodies that recognize PMK-1, MPK-1 and Actin (loading control). Worms at the L4 stage were cultured on plates containing live *E. coli* OP50 for approximately 48 h and lysates were prepared. The blot is typical of three independent experiments. *, P < 0.05; **, P < 0.01; ***, P < 0.001; ****, P < 0.0001. Two-tailed Student’s t-test. Error bars represent SEM.


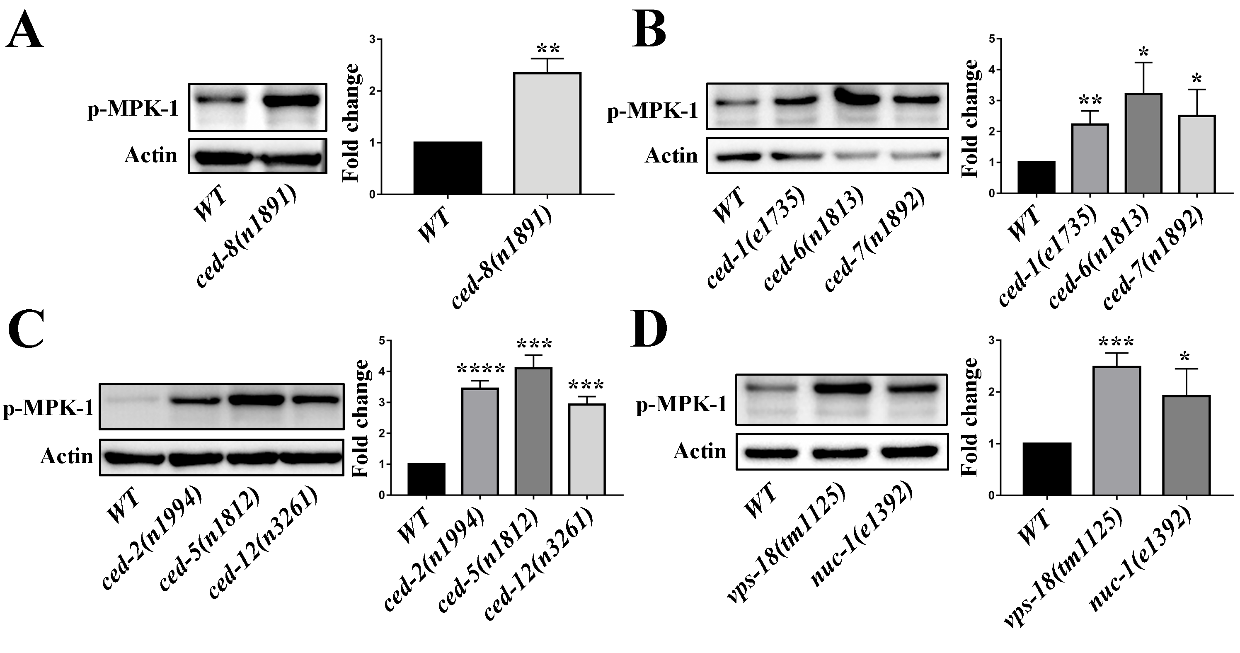


Figure S6 Defective clearance of apoptotic cell increases the level of activated MPK-1 in *C. elegans.* Related to Figure3

(A-E) Immunoblot analysis of lysates from worms of apoptotic cell clearance defective mutants and wild-type N2 (WT) using antibodies that recognize MPK-1 (p-MPK-1) and Actin (loading control). Worms at the L4 stage were cultured on plates containing live *E. coli* OP50 for approximately 48 h and lysates were prepared. The blot is typical of three independent experiments. *, *P* < 0.05; **, *P* < 0.01; ***, *P* < 0.001; ****, *P* < 0.0001. Two-tailed Student’s t-test. Error bars represent SEM.


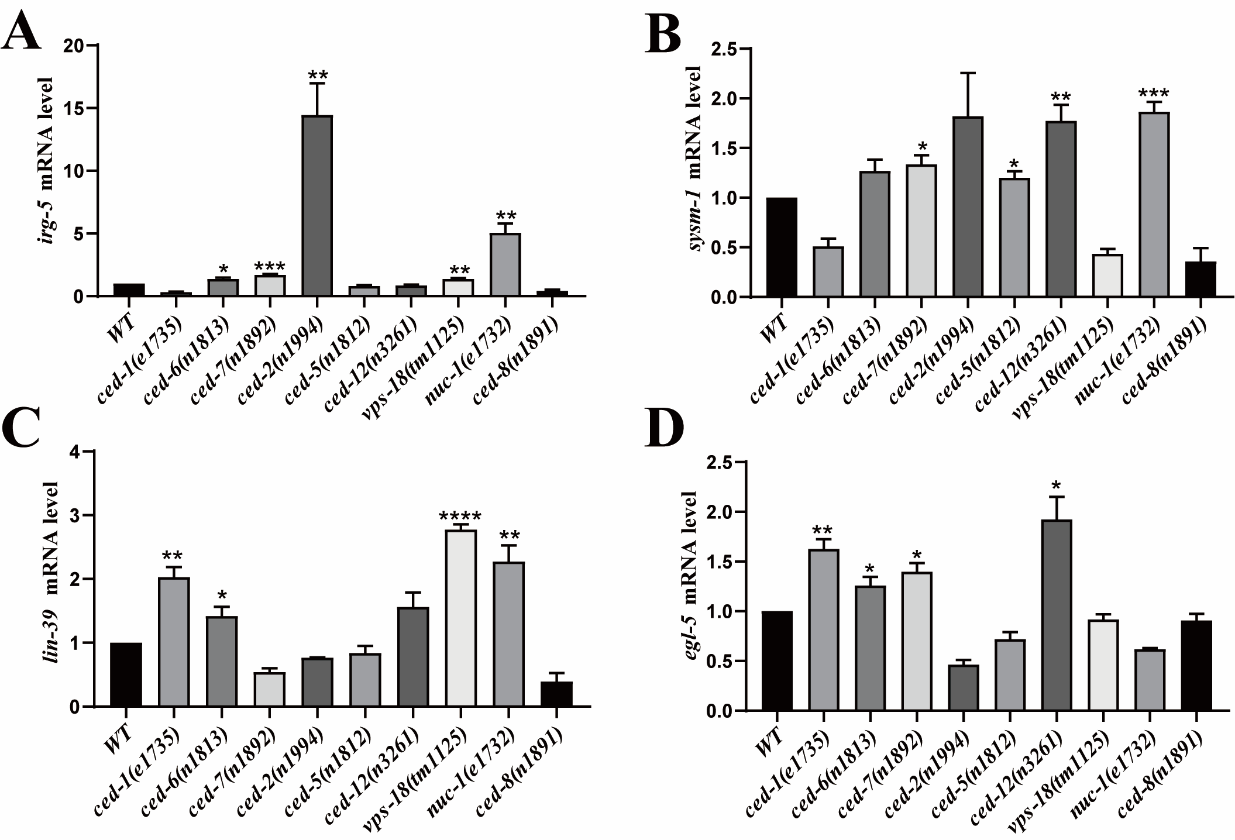


Figure S7 Increased expression of *pmk-1* and *mpk-1* dependent genes in apoptotic cell clearance mutants without bacteria pathogens infection. Related to Figure3

(A-F) Bars represent mRNA levels for *pmk-1* dependent genes *irg-5* (A), *sysm-1* (B), and *mpk-1* dependent genes *lin-39* (C), *egl-5* (D), determined by qRT-PCR in WT and apoptotic cell clearance defective mutant cultured on live OP50. Values in arbitrary units (AU) are the average of at least 3 biological replicates. *, P < 0.05; **, P < 0.01; ***, P < 0.001; ****, P < 0.0001; not significant (ns), P > 0.05. Two-tailed Student’s t-test. Error bars represent SEM.


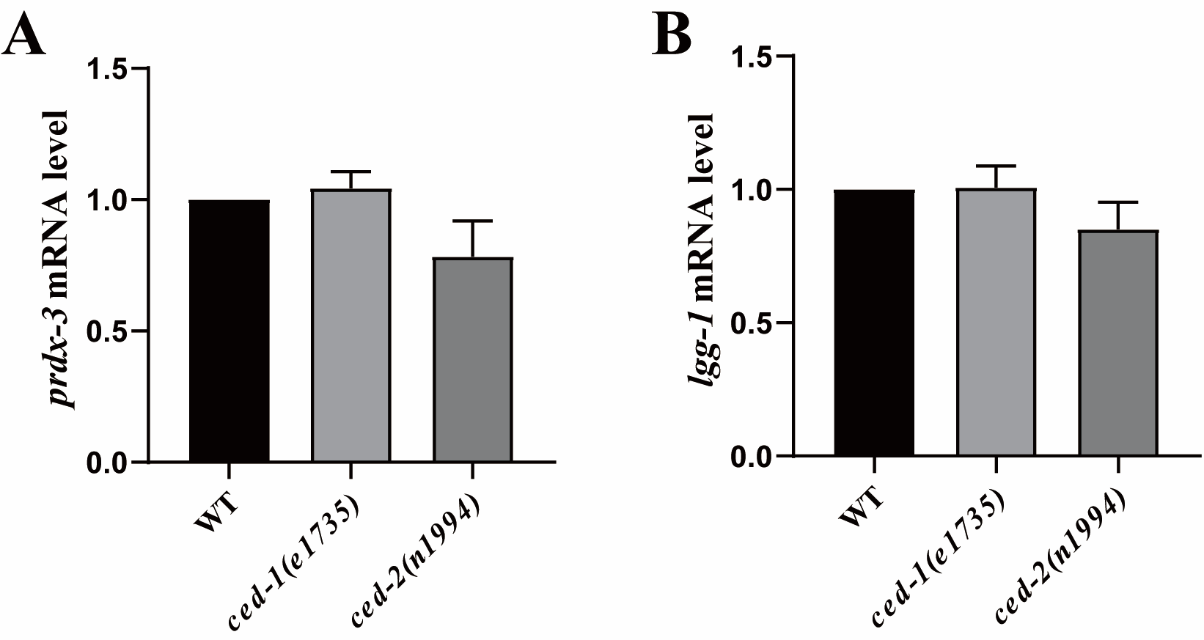


Figure S8 Defective clearance of apoptotic cell have no effect on the expression levels of endogenous targets of DAF-16 pathway. Related to Figure3

(A) Bars represent mRNA levels for *daf-16* dependent genes *prdx-3* and (B) *lgg-1*, determined by qRT-PCR in WT and apoptotic cell clearance defective mutant cultured on live OP50. Values in arbitrary units (AU) are the average of at least 3 biological replicates.


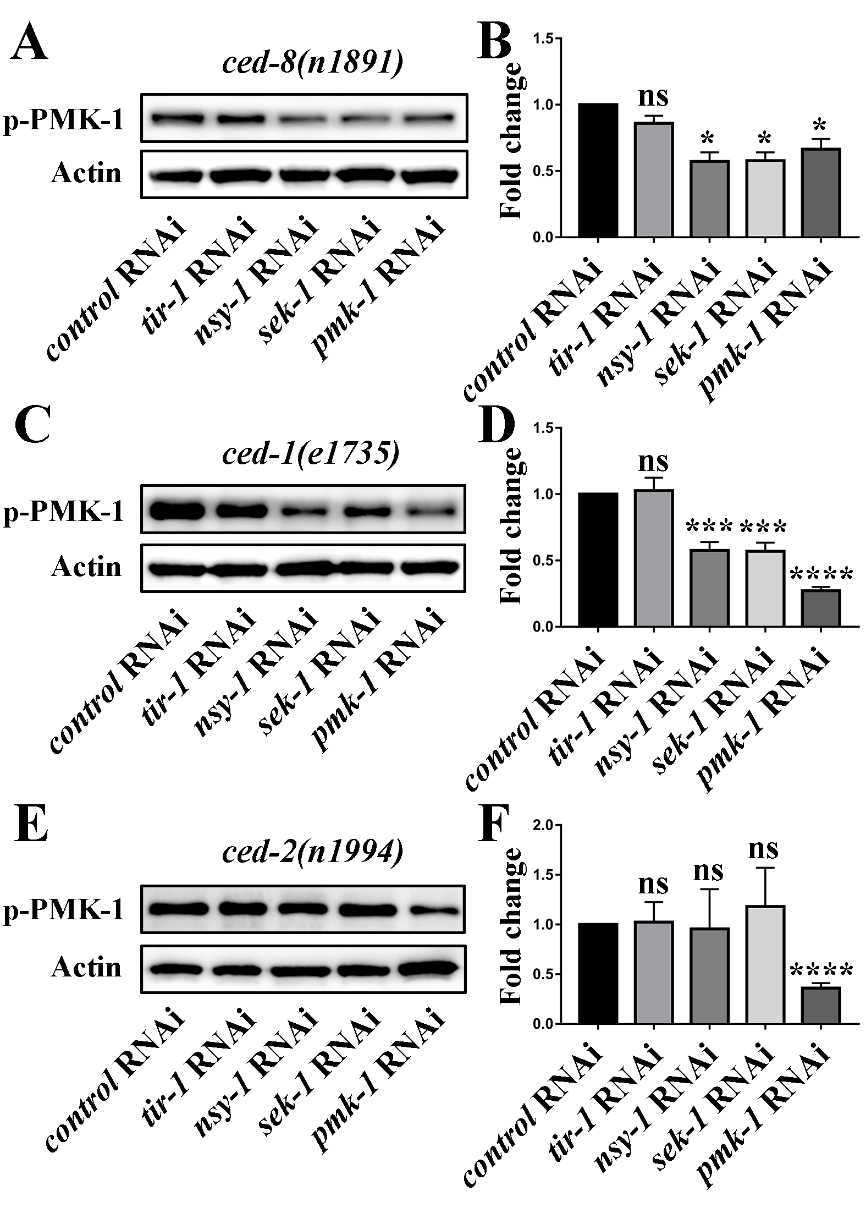


Figure S9 Genetic epistasis analysis of *ced-8*, *ced-1* and *ced-2* genes on PMK-1 p38 MAPK signaling pathway in *C. elegans.* Related to Figure 4

(A, C, E) Immunoblot analysis of lysates from *ced-8(n1891)* (A), *ced-1(e1735)* (C) and *ced-2(n1994)* (E) mutant worms using antibodies that recognize PMK-1 (p-PMK-1) and Actin (loading control). The worms were cultured on plates containing *129.36* RNAi, *tir-1* RNAi, *nsy-1* RNAi, *sek-1* RNAi, and *pmk-1* RNAi bacteria for approximately 48 h after the L4 stage and lysates were prepared. The blot is typical of three independent experiments. (B, D, F) Quantitative analysis of immunoblot test results. *, *P* < 0.05; ***, *P* < 0.001; ****, *P* < 0.0001; ns, *P* > 0.05. Two-tailed Student’s t-test. The error bars represent SEM

Table S1 Lifespan of *C. elegans* N2 and apoptotic cell clearance defective mutants with growth on *P. aeruginosa* PA14

| Genotype | TD_50_ (Mean ± SEM)（h） | *P* value |
| --- | --- | --- |
| N2  *ced-1(e1735)*  *ced-6(n1813)*  *ced-7(n1892)*  *ced-2(n1994)*  *ced-5(n1812)*  *ced-12(n3261)*  *vps-18(tm1125)*  *nuc-1(e1392)*  *ced-8(n1891)*  *pmk-1(km25)* | 75.11 ± 1.974  93.14 ± 7.682**  89.55 ± 4.15*  96.76 ± 8.607**  121.4 ± 7.099****  108.4 ± 7.026****  97.28 ± 9.277**  96.63 ± 3.644***  113.6 ± 10.98****  87.78 ± 3.158*  35.28 ± 2.742**** | —  0.0047  0.0113  0.0014  < 0.0001  < 0.0001  0.0014  0.0005  < 0.0001  0.0216  < 0.0001 |

Worms were synchronized by treating adult hermaphrodites with alkaline hypochlorite. Worms were fed the live *E. coli* OP50 and transferred at the L4 stage to plates containing *P. aeruginosa* PA14 bacteria and incubated at 25 °C. The L4 stage was designated as day 0. A total of 100 worms were quantified in each strain. Two-tailed student's t-test method was performed to compare all the other datasets with wild type (WT). All lifespan assays were repeated at least three times. *, *P* < 0.05; **, *P* < 0.01; ***, *P* < 0.001; ****, *P* < 0.0001.

Table S2 Lifespan of *C. elegans* N2 and apoptotic cell clearance defective mutants with growth on *S. typhimurium* SL1344

| Genotype | TD_50_ (Mean ± SEM)（d） | *P* value |
| --- | --- | --- |
| N2  *ced-1(e1735)*  *ced-6(n1813)*  *ced-7(n1892)*  *ced-2(n1994)*  *ced-5(n1812)*  *ced-12(n3261)*  *vps-18(tm1125)*  *nuc-1(e1392)*  *ced-8(n1891)*  *pmk-1(km25)* | 12.15 ± 0.2998  13.81 ± 0.7309*  14.45 ± 0.9374**  15.19 ± 1.136**  14.29 ± 1.184*  14.17 ± 0.9961*  14.08 ± 0.4132*  13.93 ± 0.9127*  14.74 ± 0.609**  15.74 ± 0.6826***  10.02 ± 0.5188** | —  0.0332  0.0085  0.0021  0.0200  0.0190  0.0101  0.0313  0.0020  0.0002  0.0064 |

Worms were synchronized by treating adult hermaphrodites with alkaline hypochlorite. Worms were fed the live *E. coli* OP50 and transferred at the L4 stage to plates containing *S. typhimurium* SL1344 bacteria and incubated at 20 °C. The L4 stage was designated as day 0. A total of 100 worms were quantified in each strain. Two-tailed student's t-test method was performed to compare all the other datasets with wild type (WT). All lifespan assays were repeated at least three times. *, *P* < 0.05; **, *P* < 0.01; ***, *P* < 0.001.

Table S3 Lifespan changes of *ced-1(e1735)* and *ced-2(n1994)* mutants treated with RNAi bacteria infection by *P. aeruginosa* PA14

| **Genotype** | **TD_50_(Mean ± SEM)（h）** | ***P* value** |
| --- | --- | --- |
| N2/*pmk-1* RNAi | 57.24 ± 1.56 | — |
| *ced-1(e1735)*/*pmk-1* RNAi | 62.56 ± 1.021* | 0.0462 |
| *ced-1(e1735)*/*control* RNAi | 79.34 ± 1.37 | — |
| *ced-1(e1735)*/*pmk-1* RNAi | 62.56 ± 1.021*** | 0.0006 |
| N2/*pmk-1* RNAi | 57.24 ± 1.56 | — |
| *ced-2(n1994)*/*pmk-1* RNAi | 75.04 ± 2.48** | 0.0037 |
| *ced-2(n1994)*/*control* RNAi | 93.98 ± 1.96 | — |
| *ced-2(n1994)*/*pmk-1* RNAi | 75.04 ± 2.48** | 0.0039 |

A total of 100 worms were quantified in each strain. Two-tailed student's t-test method was performed to compare all the other datasets with control group. All lifespan assays were repeated at least three times. *, *P* < 0.05.

Table S4 Lifespan changes of *ced-1(e1735)* and *ced-2(n1994)* mutants treated with RNAi bacteria infection by *P. aeruginosa* PA14

| **Genotype** | **TD_50_(Mean ± SEM)（h）** | ***P* value** |
| --- | --- | --- |
| N2/*mpk-1* RNAi | 71.84 ± 2.578 | — |
| *ced-1(e1735)*/*mpk-1* RNAi | 80.59 ± 1.011* | 0.0342 |
| *ced-1(e1735)*/*control* RNAi | 88.74 ± 2.609 | — |
| *ced-1(e1735)*/*mpk-1* RNAi | 80.59 ± 1.011* | 0.0435 |
| N2/*mpk-1* RNAi | 69.52 ± 2.093 | — |
| *ced-2(n1994)*/*mpk-1* RNAi | 80.85 ± 2.828* | 0.0181 |
| *ced-2(n1994)*/*control* RNAi | 96.20 ± 5.408 | — |
| *ced-2(n1994)*/*mpk-1* RNAi | 80.85 ± 2.828* | 0.0455 |

A total of 100 worms were quantified in each strain. Two-tailed student's t-test method was performed to compare all the other datasets with control group. All lifespan assays were repeated at least three times. *, *P* < 0.05.

Table S5 Lifespan changes of *ced-1(e1735)* and *ced-2(n1994)* mutants treated with RNAi bacteria infection by *P. aeruginosa* PA14

| **Genotype** | **TD_50_(Mean ± SEM)（h）** | | ***P* value** |
| --- | --- | --- | --- |
| N2/*pmk-1*+*mpk-1* RNAi | 57.20 ±1.526 | — | |
| *ced-1(e1735)*/ *pmk-1*+*mpk-1* RNAi | 70.59 ±0.5237** | 0.0012 | |
| *ced-1(e1735)*/*control* RNAi | 81.50 ±1.911 | — | |
| *ced-1(e1735)*/ *pmk-1*+*mpk-1* RNAi | 70.59 ±0.5237** | 0.0053 | |
| N2/ *pmk-1*+*mpk-1* RNAi | 57.20 ±1.526 | — | |
| *ced-2(n1994)*/ *pmk-1*+*mpk-1* RNAi | 71.60 ±0.9016** | 0.0012 | |
| *ced-2(n1994)*/*control* RNAi | 81.21 ±2.021 | — | |
| *ced-2(n1994)*/ *pmk-1*+*mpk-1* RNAi | 71.60 ±0.9016* | 0.0122 | |

A total of 100 worms were quantified in each strain. Two-tailed student's t-test method was performed to compare all the other datasets with control group. All lifespan assays were repeated at least three times. *, *P* < 0.05,**, *P* < 0.01; ***, *P* < 0.001..

Table S6 The primers used in qRT-PCR

| *tbg-1* Forward | CGTCATCAGCCTGGTAGAACA | This study |
| --- | --- | --- |
| *tbg-1* Reverse | TGATGACTGTCCACGTTGGA | This study |
| *nlp-29* Forward | TCCTTCTCGCCTGCTTCA | This study |
| *nlp-29* Reverse | CTTTCCCCATCCTCCATACA | This study |
| *clec-7* Forward | GGCCGGCTTCAAATGTTTATC | This study |
| *clec-7* Reverse | TAGTGGACATTACCATGCAGTC | This study |
| *clec-60* Forward | CTGAGCCAAGAACCACAAGA | This study |
| *clec-60* Reverse | GAAGTGCTGACTGACGAAAGA | This study |
| *clec-82* Forward | TTCCGCCGTTGTCTGTTT | This study |
| *clec-82* Reverse | CACTTGAGCTGGCTAGATTGA | This study |
| *lys-5* Forward | CGGGAAGTGTAGATACTGTTGG | This study |
| *lys-5* Reverse | AGAGACGCCTTAACTTGGTTAG | This study |
| *F53A9.8* Forward | GTTCACCATGCAGGAGATCA | This study |
| *F53A9.8* Reverse | TCTCCATCTTGGTGTTGAGTTT | This study |
| *irg-5* Forward | GGTACACAATCATTTGCGATGG | This study |
| *irg-5* Reverse | GCTTGGAATCTGAACTTTGGTG | This study |
| *sysm-1* Forward | ACCGATACTCCATTTGTATGCG | This study |
| *sysm-1* Reverse | GTGCAGTTGTACCACAGATTTG | This study |
| *lin-39* Forward | TGACAAGAAAGGCATCAGTGG | This study |
| *lin-39* Reverse | CCTTGTGTATGCTGTTCGTTG | This study |
| *egl-5* Forward | CTTCACAACCAGATGCCAATG | This study |
| *egl-5* Reverse | CATGTACGCAGCCGAGATAC | This study |
| *lgg-1* Forward | CTTTTGTCACTTCGTCCACTTG | This study |
| *lgg-1* Reverse | CGAGCCATTTTCCTTTGTAGTC | This study |
| *prdx-3* Forward | GGAACTGTCCGTCACACTAC | This study |
| *prdx-3* Reverse | TGGCTTGATGGTTGGAGAATC | This study |

**Supplementary Materials and methods**

***C. elegans* and bacterial strains:** *C. elegans* maintenance was performed using standard protocols [25]. Strains were grown at 20 ℃ on NGM agar plates seeded with *E. coli* OP50, unless otherwise indicated. N2 was used as the wild-type strain. Mutated and transgenic strains used in this study include the following: *ced-1(e1735)*, *ced-6(n1813)*, *ced-7(n1892)*, *ced-2(n1994)*, *ced-5(n1812)*, *ced-12(n3261)*, *ced-8(n1891)*, *vps-18(tm1125)*, *nuc-1(e1994)*, *pmk-1(km25)*, *ced-1(e1735);ced-2(n1994),* HMZ009. *E. coli* OP50, *P. aeruginosa* PA14 and *S. typhimurium* SL1344 were used in this study.

**RNA interference (RNAi):** RNA interference was performed by feeding *C. elegans* with RNAi bacteria that express double-stranded RNA (dsRNA) targeting the gene of interest. 129.36 RNAi, *ced-1* RNAi, *ced-2* RNAi, *nsy-1* RNAi, *pmk-1* RNAi, *mpk-1* RNAi were obtained from the RNAi library. The *tir-1* RNAi and *sek-1* RNAi were provided by our laboratory. All RNAi clones were verified by DNA sequencing. For RNAi experiments, single colonies of RNAi bacteria were isolated from Luria-Bertani (LB) dishes containing 50 μg/ml ampicillin, and overnight cultures of RNAi bacteria were grown in LB liquid culture medium containing 50 μg/ml ampicillin. The starter cultures were used to inoculate larger cultures at 1:100 dilution in LB liquid culture medium containing 50 μg/ml ampicillin and grown for approximately 4 h at 37 ℃. RNAi bacteria were spotted onto NGM agar plates containing 1 mM IPTG and 50 μg/ml ampicillin. The seeded plates were incubated for approximately 2 days at room temperature before worms were transferred to them. Eggs were isolated by treating adult hermaphrodites with alkaline hypochlorite and allowed to develop and grow on specific RNAi-expressing bacterial strains before lifespan and immunoblot analysis.

**Lifespan assays:** Lifespan assays by *P. aeruginosa* PA14 infection were conducted at 25 ℃ [26]. Lifespan assays by *S. typhimurium* SL1344 infection were conducted at 20 ℃ [17]. A total of 100 worms were quantified in each lifespan assay, and all experiments were repeated at least three times. A single colony of *P. aeruginosa* PA14 or *S. typhimurium* SL1344 was inoculated into 3 ml of LB medium, and allowed to incubate at 37 ℃ for approximately 12 h; 10 μl of this culture was added to 60 mm tissue culture plates containing 4 ml of NGM agar plates. Plates containing *P. aeruginosa* PA14 were incubated for 24 h at 37 ℃, and 24 h at 25 ℃. Plates containing *S. typhimurium* SL1344 were incubated for 48 h at room temperature. A total of 100 worms at the L4 stage were picked to each of the five assay plates per experimental condition. The L4 stage was designated as day 0, and worms were transferred every 1-2 days to fresh plates to eliminate overcrowding by progeny and until they laid no further eggs. Worms were considered dead when they no longer responded to touch with a platinum wire and stopped pharyngeal pumping. Worms that died of bursting the vulva or crawling onto the wall of the culture plate were eliminated from the analysis. The survival curve was plotted using GraphPad Prism 8. Each mutant's mean lifespans were compared to that for the wild type using the two-tailed student’s t-test.

**Real-time quantitative RT-PCR (qRT-PCR) assays:** Eggs were isolated by treating adult hermaphrodites with alkaline hypochlorite and spread on NGM dishes containing live *E. coli* OP50. Eggs were allowed to develop and grow until approximately 48 h after the L4 stage. These synchronized adults were washed and collected, and then total RNA was extracted using the Trizol method. The RNA was quantified using a NanoDrop and diluted in nuclease-free water. cDNA was synthesized using Transcriptor First Strand cDNA Synthesis Kit (Roche). Quantitative real-time PCR was performed using a StepOne Plus Real-Time PCR system and SYBR qPCR Master Mix (Vazyme). Using the comparative CT method, mRNA fold change was calculated by comparing mRNA levels of the gene of interest with mRNA levels of the reference gene *tbg-1*. For every gene, at least three biological replicates, each with three technical replicates. The primers used in this study are listed in Supplementary Table 5.

**Fluorescence microscopy:** For imaging fluorescence in worms, HMZ009 transgenic worms carrying *pnlp-29::gfp* and *pcol-12::dsRed* reporter genes were mounted onto 2% agar pads, paralyzed with levamisole. The slides were viewed using fluorescence microscope (Zeiss). The images were taken under the same exposure and subjected to the same processing and analysis. ImageJ was used to quantify the fluorescence intensity of P*_nlp-29_*::GFP.

**Immunoblot analyses:** Protein quality was determined by Immunoblot analysis. Worms at the L4 stage were cultured on plates containing live *E. coli* OP50 or specified RNAi bacteria for approximately 48 h, then collect them and washed twice with M9 buffer. Followed by lysis with RIPA buffer (100 mM Tris-HCl, 150 mM NaCl, 1% Triton X-100, 0.1% SDS, pH 7.2) supplemented with protease and phosphatase inhibitors on ice. And allowed to stand on ice for 30 min and centrifuged at 20000×g for 30 min at 4 °C. The supernatant was collected and protein concentration was measured by the BCA assay. 50 μg of total protein lysates were loaded into each well and separated on a 10% SDS-PAGE protein gel. The proteins were then transferred to PVDF membranes. The membranes were blocked with 5% milk or BSA for 1 h at room temperature and overnight incubated with primary antibodies. Then the membranes were incubated with species-specific HRP-conjugated secondary antibodies and incubated with chemiluminescent substrate and imaged, which were visualized using the imaging system. The band intensities of proteins were analyzed using ImageJ. The primary antibodies were anti-GFP (mouse mAb, 1:1000 dilution), anti-Phospho-p38 (rabbit mAb, 1:1000 dilution), anti-Phospho-p44/42 (rabbit mAb, 1:1000 dilution) and anti-actin antibodies (mouse mAb, 1:1000 dilution), anti-p38 MAPK (rabbit mAb, 1:1000 dilution, CST) , anti-p44/42 MAPK(Erk1/2) (mouse mAb, 1:1000 dilution, CST). The secondary antibodies were HRP-conjugated anti-mouse IgG (1:10000 dilution) and anti-rabbit IgG (1:10000 dilution).

**Colony-forming units:** To determine the number of live bacteria eaten by worms, N2, *ced-1(e1735), ced-2(n1994), ced-8(n1891)* L4 animals were fed on PA14 for 1 day at 20°C and then transferred to E. coli OP50 bacterial plates for another 3 days. The worms were washed from the plate with M9, then soaked in 25mM levamisole for 30 minutes and washed three times with M9. The worms were then soaked in M9 containing 1mg/ml ampicillin and 25mM levamisole at room temperature for 2 hours, and then washed three times with M9 containing 25mM levamisole. Ten worms were randomly selected and placed in a 1.5 mL centrifuge tube containing 20ul M9. The worms were ground using an electric grinder. The ground worms were gradient dilution coated on LB medium containing 1mg/ml ampicillin and colony count was carried out the next day.
